# Experimental Investigation Examining the Effects of Acute Exercise on Implicit Memory Function

**DOI:** 10.5964/ejop.v15i4.1837

**Published:** 2019-12-19

**Authors:** Paul D. Loprinzi, Morgan Gilbert, Gina Robinson, Briahna Dickerson

**Affiliations:** a Exercise & Memory Laboratory, Department of Health, Exercise Science and Recreation Management, The University of Mississippi, Oxford, MS, USA; University of South Wales, Pontypridd, UK

**Keywords:** consciousness, declarative memory system, physical activity, procedural memory

## Abstract

Emerging work suggests that acute exercise can enhance explicit memory function. Minimal research, however, has examined whether acute exercise is associated with implicit memory, which was the purpose of this study. Three separate experimental studies were computed (N = 120; Mean age = 21). In Experiment 1, participants were randomly assigned to either a moderate-intensity bout of acute exercise (15-minute) or engaged in a seated control task (15-minute), followed by the completion of a word-fragmentation implicit memory task. Experiment 2 replicated Experiment 1, but instead employed a higher-intensity exercise protocol. For Experiment 3, participants were randomly assigned to either a moderate-intensity bout of acute exercise (15-minute) or engaged in a seated control task (15-minute), followed by the completion of a real world, 3-dimensional implicit memory task. For Experiment 1, the exercise and control groups, respectively, had an implicit memory score of 7.0 (0.5) and 7.5 (0.6) (t(38) = 0.67, p = .51). For Experiment 2, the exercise and control groups, respectively, had an implicit memory score of 6.9 (1.9) and 7.8 (2.4) (t(38) = 1.27, p = .21). These findings suggest that exercise, and the intensity of exercise, does not alter implicit memory from a word fragmentation task. For Experiment 3, the exercise and control groups, respectively, had a discrimination implicit memory index score of 0.48 (0.18) and 0.29 (0.32) (t(38) = 2.16, p = .03). In conclusion, acute exercise does not influence a commonly used laboratory-based assessment of implicit memory but may enhance real world-related implicit memory function.

Research demonstrates that health behaviors, such as exercise, may not only have cardiometabolic protective effects, but may also favorably influence neurological function (Chang, Labban, Gapin, & Etnier, 2012; Crush & Loprinzi, 2017; Etnier et al., 2016; Frith, Sng, & Loprinzi, 2017; Labban & Etnier, 2011; Loprinzi, Frith, Edwards, Sng, & Ashpole, 2018a; Loprinzi, Herod, Cardinal, & Noakes, 2013; Loprinzi & Kane, 2015; McMorris, 2016; McMorris, Sproule, Turner, & Hale, 2011; McMorris, Turner, Hale, & Sproule, 2016; Roig, Nordbrandt, Geertsen, & Nielsen, 2013; Roig et al., 2016). Notably, these findings have been observed even among young adults, which is important as memory function may start to decline in young adulthood (Salthouse, 2009). We have previously detailed the dearth of research on this topic among this population (young adults) and have noted that the majority of this research has focused on episodic memory function (Loprinzi et al., 2018a). Episodic memory is considered the conscious recall of past events or episodes from a spatial-temporal context (Loprinzi, Edwards, & Frith, 2017). The mechanisms through which exercise may influence episodic memory function has been extensively detailed (Bherer, Erickson, & Liu-Ambrose, 2013; Loprinzi et al., 2017; McMorris, 2016; McMorris et al., 2016; Piepmeier & Etnier, 2015).

As we have recently discussed (Loprinzi & Edwards, 2017), a major gap in the literature is whether exercise can influence implicit memory function, which includes the recall of information that was not consciously encoded (Slotnick, 2016). As we have previously illustrated in a systematic review (Loprinzi & Edwards, 2017), only 10 published experiments have examined the effects of exercise on implicit memory, and among these, seven were conducted in animal models. Thus, three (Eich & Metcalfe, 2009; Padilla, Mayas, Ballesteros, & Andres, 2016; Sherman, Buckley, Baena, & Ryan, 2016) human experimental studies have examined the effects of exercise on implicit memory, with the findings of these studies being mixed (Loprinzi & Edwards, 2017).

Previous work (Barco, Bailey, & Kandel, 2006; Hawkins, Kandel, & Bailey, 2006) highlights common underlying mechanisms of explicit and implicit memory function, with both memory outcomes likely influenced, in part, from alterations in long-term potentiation, or the functional connectivity of communicating neurons. We have recently discussed the potential role through which acute exercise may induce long-term potentiation (Loprinzi et al., 2017; Loprinzi, Ponce, & Frith, 2018b), likely through exercise-induced muscle spindle and vagus nerve stimulation. Thus, mechanistically, it is biologically plausible that acute exercise may influence both explicit and implicit memory.

Couched within the above, the purpose of this study was to experimentally examine the effects of acute exercise on implicit memory function. We address this question (i.e., does acute exercise influence implicit memory?) from three sequential experimental studies conducted in our laboratory. For these three sequential experimental studies, data was collected over an 18-month period. The first experiment evaluates the effects of acute lower-intensity exercise on implicit memory, whereas the second experiment evaluates the effects of acute higher-intensity exercise on implicit memory. Both of these experimental studies employ a commonly-used laboratory assessment of implicit memory (word fragmentation/completion task). In our third experimental study, we evaluate the effects of acute moderate-intensity exercise on implicit memory while employing a more real-world, 3-dimensional assessment of implicit memory.

## Experiment 1

### Method

#### Study Design

A two-arm, parallel-group randomized controlled experiment was employed. This study was approved by the ethics committee at the University of Mississippi and participants provided written informed consent prior to participation. Participants were randomized into one of two groups (experimental and control). The experimental group walked briskly for 15 minutes, while the control group engaged in a seated, time-matched computer task (Sudoku) for 20-minutes. After the exercise condition, participants sat for 5-minutes (playing Sudoku), then completed an affect survey (for “priming” purposes), then watched a video for 5-minutes, and then completed the word completion task (implicit memory assessment).

#### Participants

Each group included 20 participants (*N* = 40). This is based from a power analysis indicating sample sizes ranging from 15 to 24 (*d*, 0.84–1.36; two-tailed α error probability, .05; 1-β error probability, .80; allocation ratio, 1). This also aligns with our related experimental work demonstrating adequate statistical power (Crush & Loprinzi, 2017; Frith et al., 2017; Loprinzi & Kane, 2015; Sng, Frith, & Loprinzi, 2017). Participants were recruited via a convenience-based, non-probability sampling approach. Participants (students) were eligible if they were between the ages of 18 and 35 years. Further, participants were excluded if they:

- Self-reported as a daily smoker (Jubelt et al., 2008; Klaming, Annese, Veltman, & Comijs, 2016)
- Self-reported being pregnant (Henry & Rendell, 2007)
- Exercised within 5 hours of testing (Labban & Etnier, 2011)
- Consumed caffeine within 3 hours of testing (Sherman et al., 2016)
- Had a concussion or head trauma within the past 30 days (Wammes, Good, & Fernandes, 2017)
- Took marijuana or other illegal drugs within the past 30 days (Hindocha, Freeman, Xia, Shaban, & Curran, 2017)
- Were considered a daily alcohol user (>30 drinks/month for women; >60 drinks/month for men) (Le Berre, Fama, & Sullivan, 2017)

#### Exercise Protocol

Those randomized to the exercise group walked on a treadmill for 15 minutes at a self-selected “brisk walk.” They were asked to select a pace as if they were late for class or to catch a bus, with a minimum speed set of 3.0 mph. This exercise protocol has been shown to enhance explicit memory function (Sng, Frith, & Loprinzi, 2018). The bout of exercise occurred prior to the memory assessment. Immediately after the bout of exercise, participants rested in a seated position for 5 minutes (played on-line Sudoku puzzle during this resting period). After this resting period, they completed a brief affective assessment asking them to rate (1, very unpleasant; 5, very pleasant) 25 different words (e.g., kids, outside), with these words employed in previous implicit memory experiments (Anderson, Carnagey, & Eubanks, 2003; Carnagey & Anderson, 2005).

After completing this brief assessment, participants watched a 5-minute video clip (The Office Bloopers Season 4). They were instructed to focus closely on this video, as after the video, they would be asked to write down three funny things from the video. The purpose of this video was to create a delay between the implicit memory prime (indicating how the words made them feel) and the word completion task. After the video, participants completed a 50-item word completion task, of which 25 words (randomly sorted) came from the affective assessment they previously completed.

#### Control Protocol

Those randomized to the control group completed the same protocol as those in the exercise group, with the exception of, instead of exercising for 15-minutes followed by resting for 5-minutes, they completed a medium-level, on-line administered, Sudoku puzzle for 20-minutes. The website for this puzzle is located here: https://www.websudoku.com/

#### Memory Assessment

Implicit memory was assessed using a 50-item word completion task, which has demonstrated evidence of both reliability (Ojemann et al., 1998) and validity (Spaan & Raaijmakers, 2011). Twenty-five words from the work of Anderson et al. (2003; Carnagey & Anderson, 2005) were used for this study. Of these 50 word fragments, 25 of the words were the words from the memory prime (affective assessment). The implicit memory outcome was the number of correctly completed words from the 25 primed words.

For each of the 50 word fragments, participants were asked to complete the word with whatever word first came to mind. As an example, one of the “primed” words (from the affective assessment) was “kids,” and the word fragment was “K I _ _.” Participants could have, for example, completed the word by writing “kite,” “kiss,” “kilt,” “king,” “kids,” “kind,” “kiwi,” “kink,” or “kilo.” If, however, they wrote “kids,” then they received a point. The maximum number of points for this implicit memory, word completion task was 25, with a higher score indicative of greater implicit memory.

#### Additional Assessments

As a measure of habitual physical activity behavior, participants completed the Physical Activity Vital Signs Questionnaire, which reported time spent per week in moderate-to-vigorous physical activity (MVPA) (Ball, Joy, Gren, & Shaw, 2016). Height/weight (BMI) were measured to provide anthropometric characteristics of the sample. Lastly, before, during and after the exercise and control conditions, heart rate (chest-strapped Polar monitor, F1 model) was assessed.

#### Statistical Analysis

All statistical analyses were computed in SPSS (v. 24). An independent samples t-test was used to compare the implicit memory score across the two groups. Statistical significance was set at an alpha of .05.

### Results

Demographic and behavioral characteristics of the sample are shown in Table 1. Participants, on average, were 21 years, with the majority of the sample (75.0%) being female. The sample comprised multiple race-ethnicity groups, including 58.0% being non-Hispanic white, 30.0% non-Hispanic black, and the remaining including a multi-racial or other classification. Resting heart rate was similar between the two groups (73–75 bpm), with the heart rate increasing to 130 bpm by the end of the exercise bout.

**Table 1:** Characteristics of the Study Variables across the Three Experiments

	Experiment 1		Experiment 2		Experiment 3	
Variable	Exercise (*N* = 20)	Control (*N* = 20)	Exercise (*N* = 20)	Control (*N* = 20)	Exercise (*N* = 20)	Control (*N* = 20)
Age, mean years	21.2 (2.2)	21.1 (2.1)	21.3 (2.3)	21.0 (1.4)	21.1 (1.5)	20.9 (1.6)
% Female	66.6	84.2	70.0	70.0	68.4	76.1
Race-Ethnicity, %						
White	57.1	57.9	70.0	60.0	42.1	38.1
Black	33.3	26.3	20.0	30.0	57.9	57.1
Other	9.5	15.5	10.0	10.0	0.0	4.8
BMI, mean kg/m^2^	24.9 (5.1)	23.6 (3.5)	25.5 (5.5)	26.4 (6.0)	25.0 (5.5)	26.0 (6.6)
MVPA, mean min/week	226.5 (194.5)	138.7 (89.6)	172.8 (114.1)	174.3 (143.0)	128.8 (136.0)	131.8 (130.5)
Heart Rate, mean bpm						
Resting	73.0 (11.2)	75.2 (12.4)	81.7 (14.1)	77.8 (12.0)	77.6 (9.3)	-
Midpoint	122.5 (19.3)	77.6 (9.4)	150.8 (9.7)	-	120.9 (14.1)	-
Endpoint	130.5 (17.6)	75.8 (10.1)	150.6 (6.7)	-	121.6 (13.6)	-
Post	82.2 (12.2)	78.5 (8.9)	93.3 (11.1)	-	81.1 (10.9)	-

*Note*. BMI = Body mass index; MVPA = Moderate to vigorous physical activity. Values in parentheses are standard deviation (*SD*) estimates. -, not assessed.

**Figure 1:**
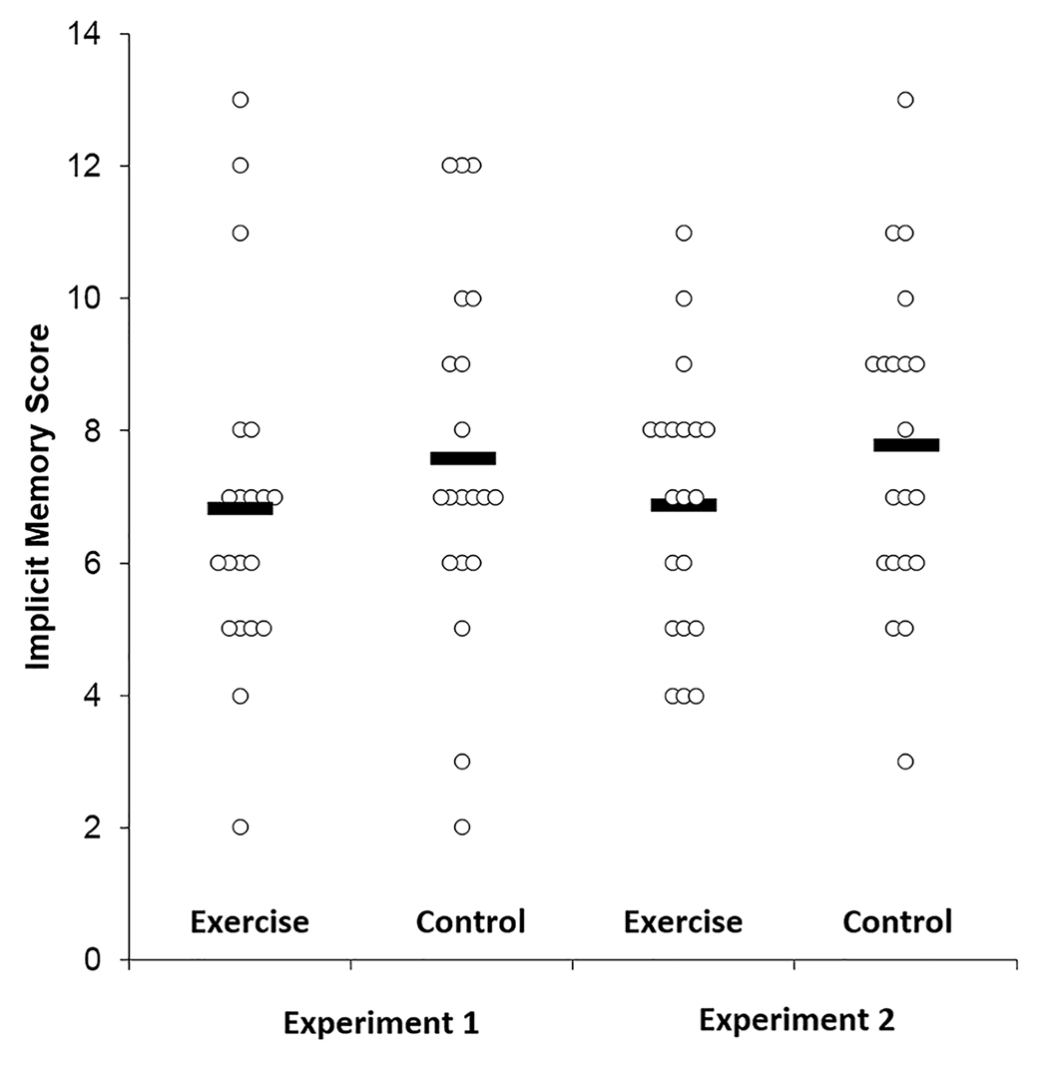
Mean and individual data points for the implicit memory task among the exercise and control groups for Experiments 1 and 2.

Figure 1 displays the results of the implicit memory assessment. The exercise and control groups, respectively, had an implicit memory score of 7.0 (0.5) and 7.5 (0.6) (*t*(38) = 0.67, *p* = .51). Thus, results from Experiment 1 showed that acute lower-intensity exercise did not influence implicit memory function. Notably, the control group had slighter higher implicit memory scores.

### Discussion

Emerging work suggests that humans can form unconscious memories (Wuethrich, Hannula, Mast, & Henke, 2018). Up until recently, the majority of experimental work in the exercise neurobiology field has examined the effects of exercise on conscious, explicit memories. Recent work has emphasized the importance of additional experiments evaluating the effects of exercise on unconscious, implicit memory (Loprinzi & Edwards, 2017). This served as the motivation for Experiment 1, which evaluated the effects of acute, lower-intensity exercise on implicit memory function. Our findings did not suggest any effect of acute exercise on implicit memory.

As noted elsewhere (Loprinzi & Edwards, 2017), only 10 published experiments have examined the effects of exercise on implicit memory, and among these, seven were conducted in animal models, employing a fear condition paradigm. Six of these seven studies employed a chronic training protocol and all six demonstrated an exercise-induced enhancement effect on implicit memory. Three (Eich & Metcalfe, 2009; Padilla et al., 2016; Sherman et al., 2016) human experimental studies, all employing a word-stem completion task, examined the effects of exercise on implicit memory. Eich and Metcalfe (2009) demonstrated that an acute marathon bout (26.2 miles) enhanced implicit memory, Sherman et al. (2016) did not demonstrate any benefits from short-duration (15-minute) sprinting, and Padilla et al. (2016) did not observe any implicit memory benefits from self-reported chronic exercise engagement.

Taken together, our findings align with the other mixed findings in this emerging line of inquiry. Previous experimental work (Chang et al., 2012; Crush & Loprinzi, 2017; Etnier et al., 2016; Frith et al., 2017; Labban & Etnier, 2011; Loprinzi et al., 2013, 2018; Loprinzi & Kane, 2015; McMorris, 2016; McMorris et al., 2011, 2016; Roig et al., 2013, 2016; Sng et al., 2017) provides suggestive evidence that acute exercise can subserve explicit memory function, likely as a result of enhanced neuronal excitability, alterations in neurotrophic factors, and augmentation of long-term potentiation, or the functional connectivity across neurons. It is conceivable that acute exercise may also facilitate implicit memory, as there are common molecular mechanisms subserving explicit and implicit memory (Barco et al., 2006; Hawkins et al., 2006). Given the paucity of research examining the effects of exercise on implicit memory, future work on this topic is warranted.

In conclusion, our findings do not demonstrate any evidence of implicit memory enhancement or impairment from an acute bout of lower-intensity exercise. Given the paucity of research on acute exercise and implicit memory, additional work in this area is needed. One such area of future inquiry is whether the effects of acute exercise and implicit memory are intensity-dependent. This assertion aligns with our recent review demonstrating that high-intensity exercise, when compared to lower intensity acute exercise, may more favorably influence explicit memory function (Loprinzi, 2018). The extent to which this may occur for implicit memory is less understood. Thus, Experiment 2 evaluates the effects of acute higher-intensity exercise on implicit memory function.

## Experiment 2

As we have recently discussed, higher-intensity exercise (vs. lower intensity acute exercise) may more favorably impact explicit memory systems (Loprinzi, 2018). For example, high-intensity acute exercise that occurs shortly before memory encoding may increase levels of select neurotransmitters (e.g., norepinephrine, dopamine, serotonin, and acetylcholine). Elevation of these neurotransmitters may activate various intracellular neuronal pathways (e.g., activate of kinases, such as Protein Kinase A), which in turn, may increase the phosphorylation of transcription factors (e.g., CREB) that subserves long-term potentiation (Loprinzi et al., 2017), a key cellular correlate of memory function (Loprinzi, 2018). Notably, some of the same underlying mechanisms that influence explicit memory may also influence implicit memory function (Barco et al., 2006; Hawkins et al., 2006).

As discussed previously, higher-intensity marathon exercise may be a potential stimulus to enhance implicit memory function (Eich & Metcalfe, 2009). However, among the few studies on this topic, other work does not demonstrate an implicit memory benefit from high-intensity exercise. That is, Sherman et al. (2016) did not demonstrate any implicit memory benefits from short-duration sprinting. These mixed findings warrant future research. In particular, a protocol that combines both aspects of these two higher-intensity protocols may be sensible and feasible (i.e., shorter duration, continuous exercise). Thus, the purpose of Experiment 2 was to evaluate the potential effects of acute higher-intensity continuous exercise on implicit memory function.

### Method

This study was approved by the ethics committee at the University of Mississippi and participants provided written informed consent prior to participation. The entire protocol for Experiment 2 was identical to Experiment 1, except for the exercise stimuli. Participants jogged on a treadmill for 15 minutes at approximately 80% of their estimated heart rate max (220-age). After this exercise bout, participants sat and played Sudoku for 10-minutes. The control group (time-matched) played Sudoku for 25-minutes.

### Results

Demographic and behavioral characteristics of the sample for Experiment 2 are also shown in Table 1. Unlike Experiment 1 (lower-intensity exercise) where heart rate increased to 130 bpm by the end of the exercise bout, for Experiment 2 (higher-intensity exercise), heart rate increased up to 150 bpm by the end of the exercise bout, which was statistically significantly higher than the exercise heart rate observed in Experiment 1 (*p* < .001). Notably, there were no differences in resting heart rate between the two experiments (*p* > .10).

Figure 1 displays the results of the implicit memory assessment for both Experiment 1 and 2. For Experiment 2, the exercise and control groups, respectively, had an implicit memory score of 6.9 (1.9) and 7.8 (2.4) (*t*(38) = 1.27, *p* = .21). Similar to Experiment 1, the control group for Experiment 2 had a slightly higher mean implicit memory score than the exercise group.

### Discussion

For both Experiment 1 (lower-intensity exercise) and Experiment 2 (higher-intensity exercise), acute exercise did not enhance implicit memory function. Although we cannot fully discount the possibility that these null effects may be a result of the exercise bout duration (15-minutes), our other experimental work demonstrates that this exercise duration (or even less) has been shown to enhance explicit memory function (Frith et al., 2017; Haynes Iv, Frith, Sng, & Loprinzi, 2018; Jaffery, Edwards, & Loprinzi, 2018; Sng et al., 2018). Of course, however, it is possible that memory type (explicit vs. implicit) may moderate the effects of exercise duration on memory.

Of interest here is whether the type of implicit memory assessment may be accounting for our observed null findings. Thus, the purpose of Experiment 3 was to employ a more real-world, 3-dimensional assessment of implicit memory, and evaluate whether acute exercise influences implicit memory when assessed in this manner.

## Experiment 3

With a few exceptions (Brewer & Treyens, 1981; Droll & Eckstein, 2009; Pezdek, Whetstone, Reynolds, Askari, & Dougherty, 1989; Qin et al., 2014), most recognition memory tasks involve the recollection of items from computerized images (i.e., the encoding and recognition *both* occurring from computerized images). Although this approach may facilitate greater laboratory control, there is a trade-off as it may lack generalizability to real-world situations. Thus, it is important that, when feasible, laboratory assessments attempt to maximize this generalizability in a real-world context by including testing formats that include real-world, 3-D objects. Experiment 3 aims to accomplish this. That is, the aim of Experiment 3 was to examine whether acute exercise can enhance implicit memory of 3-D objects in a real world-type context.

### Method

This study was approved by the ethics committee at the University of Mississippi and participants provided written informed consent prior to participation. The entire protocol for Experiment 3 was identical to Experiment 1, with the exception of the memory assessment. A two-arm, parallel-group randomized controlled intervention was employed. Participants were randomized into one of two groups. The experimental group walked briskly for 15 minutes on a treadmill (5-minute post-exercise rest), while the control group engaged in a 20-minute seated task. After this, the implicit memory task was commenced.

#### Memory Assessment and Procedures

**Figure 2:**
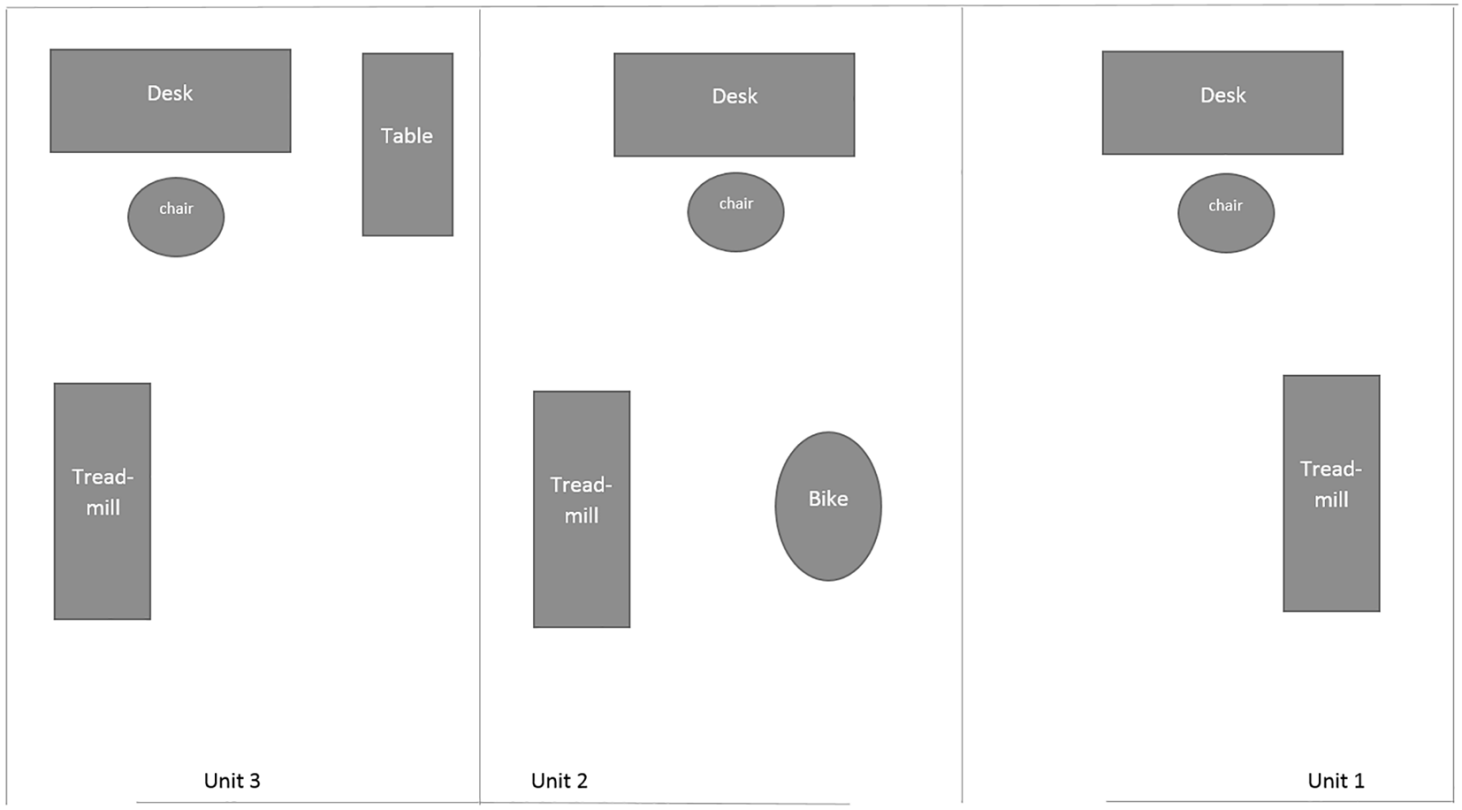
Schematic overview of the laboratory set-up for Experiment 3.

The laboratory set-up included three adjacent 10’ × 10’ rooms, each separated by a wall (6’ in height). See Figure 2 for a schematic of the laboratory set-up. Participants entered the laboratory through the door entrance, which is near Unit 1 (Figure 2). They sat in the chair in Unit 1 to complete the consent document and demographic survey. At this time, the participant were asked to give the researcher their cellphone, which was returned to the participant after the study was completed. This was performed to ensure that the participant did not distract themselves with their phone during the experimental protocol.

After going over the consent document and demographic survey, participants then exercised for 15-minutes on the treadmill in Unit 1 (if in the exercise group). After this bout of exercise, the participant sat back in the chair in Unit 1 for 5 minutes. During this 5-minute resting period, they sat quietly and completed a medium-level, on-line administered, Sudoku puzzle (same as the control group).

After this 5-minute resting period, the participant got up from the chair in Unit 1 and walked over to Unit 3 and sat in the chair in front of the desk. They were instructed to sit in the chair quietly for 2-minutes. They were not given any tasks during this period and were told to sit quietly for a few minutes. The computer at their desk was turned off, so they were not be able to be distracted by any electronic device (e.g., computer or cell phone). After this 2-minute period, the researcher signaled them to come back to Unit 1. When returning to the chair in Unit 1, they completed another on-line Sudoku puzzle for 5-minutes. After this, they completed the incidental memory task.

The incidental memory task was completed on the computer in Unit 1. They observed a series of 18 objects on the computer, with one object appearing per slide. They advanced through the 18 slides at a self-directed pace. For each image, they were asked to indicate if they saw the image when they were in Unit 3 (completed via paper-and-pencil). They were told to focus their attention on the object, rather than its appearance (e.g., apparent size or distance). The image was a close-up picture of the object, with this picture taken in a different environment. For each image, participants selected one of three options via a paper-pencil document. The possible options include: “remember,” “know,” and “new.” See Table 2 for the description of these options.

**Table 2:** Response Options for the Implicit Recognition Memory Task (Experiment 3)

Response Option	Description
Remember	“Remember” is the ability to become consciously aware again of some aspect or aspects of what happened or what was experienced at the time you were exposed to the object (e.g., aspects of the physical appearance of the image, or something that you were thinking at the time of viewing the image). In other words, the “Remember” images should bring back to mind a particular association, image, or something more personal from the time of the study, or something about its appearance or position.
Know	“Know” responses should be made when you recognize seeing the image when in the other unit, but you cannot consciously recollect anything about its actual occurrence or what happened or what was experienced at the time of its occurrence.
New	“New” responses would be if you are certain that you did not previously see the image when in the other unit.

Nine of the 18 images that they were tested on were objects that were placed in Unit 3. These nine objects were placed on the desk they were sitting at (in Unit 3), the table near the desk, or on the floor next to the treadmill. Of the nine objects placed in Unit 3, three were objects that we classified as “context- typical object,” three were “context-atypical objects,” and three were “context-unfamiliar objects.” See Table 3 for a description of these objects. One object from each of these categories was placed on the desk, table, and floor in Unit 3. The other nine images that they viewed on the computer screen were objects that were not in Unit 3, but fall within these three categories. That is, for each object placed in the experimental room (Unit 3), we selected a similar object from the same object-type (i.e., context-typical, context-atypical, or unfamiliar). The 18 images/slides were randomly ordered.

**Table 3:** Description of the Objects Used in Experiment 3

Context-Typical Objects	Context-Atypical Objects	Context-Unfamiliar Objects
Binder (3-ring)	Spatula	Jawzrsize
Textbook	Hanger	Miniaturized HJ Scraper
Stapler	Sunscreen lotion container (8 oz)	Wrist attachment device

After the completion of the study, participants completed a brief survey confirming the categorization of these objects. These questions are shown in Table 4. Additionally, after the conclusion of the study, participants completed a brief survey assessing their anticipation of the memory task. See Table 5 for the specific questions used to evaluation their anticipation of the memory protocol.

**Table 4:** Survey Questions Assessing Participant Confidence, Familiarity, and Typicality of Each Object (Experiment 3)

Naming Confidence	Context-Typical Objects	Context-Unfamiliar Objects
“How confident are you that you know the specific name of this object.”	“How typical do you consider this object in the context of a University.”	“How often do you encounter this object in your daily live?”
1 = not at all confident; 2 = little confidence; 3 = neither; 4 = somewhat confident; 5 = very confident	1 = not typical at all; 2 = somewhat typical; 3 = neither; 4 = typical; 5 = very typical	1 = never; 2 = rarely; 3 = occasionally; 4 = very frequently; 5 = always

**Table 5:** Questions Used to Classify Anticipation of the Memory Task (Experiment 3)

Question	Response Options
“When I was in Unit 3, I suspected that I would be tested on the identities of the objects on the desk, table and/or floor.”	1 = strongly disagree; 2 = disagree; 3 = neutral; 4 = agree; and 5 = strongly agree.
“When I was in Unit 3, I made an effort to memorize the identities of the objects on the desk, table and/or floor.”	1 = strongly disagree; 2 = disagree; 3 = neutral; 4 = agree; and 5 = strongly agree.

#### Data Reduction for the Memory Task

For the memory recognition assessment, a hit rate score was calculated as a rate of correctly indicating that they previously saw the image when in Unit 3 (i.e., that they “remembered” or “knew” they saw the image). A false rate score was calculated as a rate of incorrectly indicating that they previously “remembered” or “knew” seeing an image that was not present in Unit 3. Lastly, the discrimination index was calculated as “hit rate—false rate.”

### Results

Table 1 describes the characteristics of the sample. Similar to Experiment 1, for Experiment 3, heart rate increased from the 70’s (bpm) to the 120’s (bpm).

Regarding the categorization of the objects, which, as shown in Table 3, were categorized into one of the following three categories: context-typical, context-atypical, and context-unfamiliar. Table 4 reports the survey questions that were employed to evaluate how confident they were in naming the object, how typical they consider the object in the laboratory setting, and how often they encountered the object in their daily life. Regarding their confidence ratings, the mean (standard deviation, *SD*) confidence ratings for typical, atypical, and unfamiliar objects, respectively, were 4.45 (0.55), 4.86 (0.44), and 2.20 (0.7). This confirms that our selected objects were categorized appropriately. Regarding how typical participants considered the object in the laboratory setting, the mean (*SD*) typical ratings for typical, atypical, and unfamiliar objects, respectively, were 4.0 (0.51), 2.64 (0.99), and 2.17 (0.83). This confirms that our selected objects were categorized appropriately in the context of how typical they thought the object was for this research setting. Lastly, participants indicated how often they encountered the object in their daily lives. The mean (*SD*) ratings for typical, atypical, and unfamiliar objects, respectively, were 3.18 (0.60), 3.65 (0.53), and 1.49 (0.43). This confirms that our selected objects were categorized appropriately for how often they encounter these objects in their daily life. As shown in Table 5, we asked two remaining questions at the end of the visit, including whether the participant anticipated that the objects in the room were related to the memory task and whether they attempted to memorize these objects. For both of these items (Table 4), response options ranged from 1 (strongly disagree) to 5 (strongly agree). The mean (*SD*) score for whether they anticipated that they would be asked to recall the object and whether they tried to memorize the object, respectively, were 1.62 (1.0) and 1.67 (1.0). Thus, participants “strongly disagreed” to “disagreed” that they anticipated these object were part of the memory protocol and that they tried to memorize the objects.

Table 6 displays the hit rate, false rate, and discrimination index across the exercise and control groups for the context-typical, context atypical, and context-unfamiliar items. The discrimination index was statistically significantly higher for the exercise (vs. control) group for context typical objects (0.44 vs. 0.23, *p* = .03) and for the overall discrimination index (0.48 vs. 0.29, *p* = .03).

**Table 6:** Mean (SD) Hit Rate, False rate, and Discrimination Index (d’) between the Exercise and Control Groups

Group	Context-Typical		Context-Atypical		Context-Unfamiliar				
	Hit-Rate	*d*’	Hit-Rate	*d*’	Hit-Rate	*d*’	Overall Hit Rate	Overall False Rate	Overall *d*’
**Exercise**	0.63 (0.31)	0.44 (0.26)	0.61 (0.22)	0.42 (0.17)	0.75 (0.29)	0.57 (0.31)	0.67 (0.21)	0.18 (0.16)	0.48 (0.18)
**Control**	0.57 (0.30)	0.23 (0.32)	0.65 (0.28)	0.31 (0.30)	0.68 (0.37)	0.34 (0.52)	0.63 (0.21)	0.34 (0.29)	0.29 (0.32)
***p*-value**	.53	.03	.66	.16	.50	.11	.64	.05	.03

**Figure 3:**
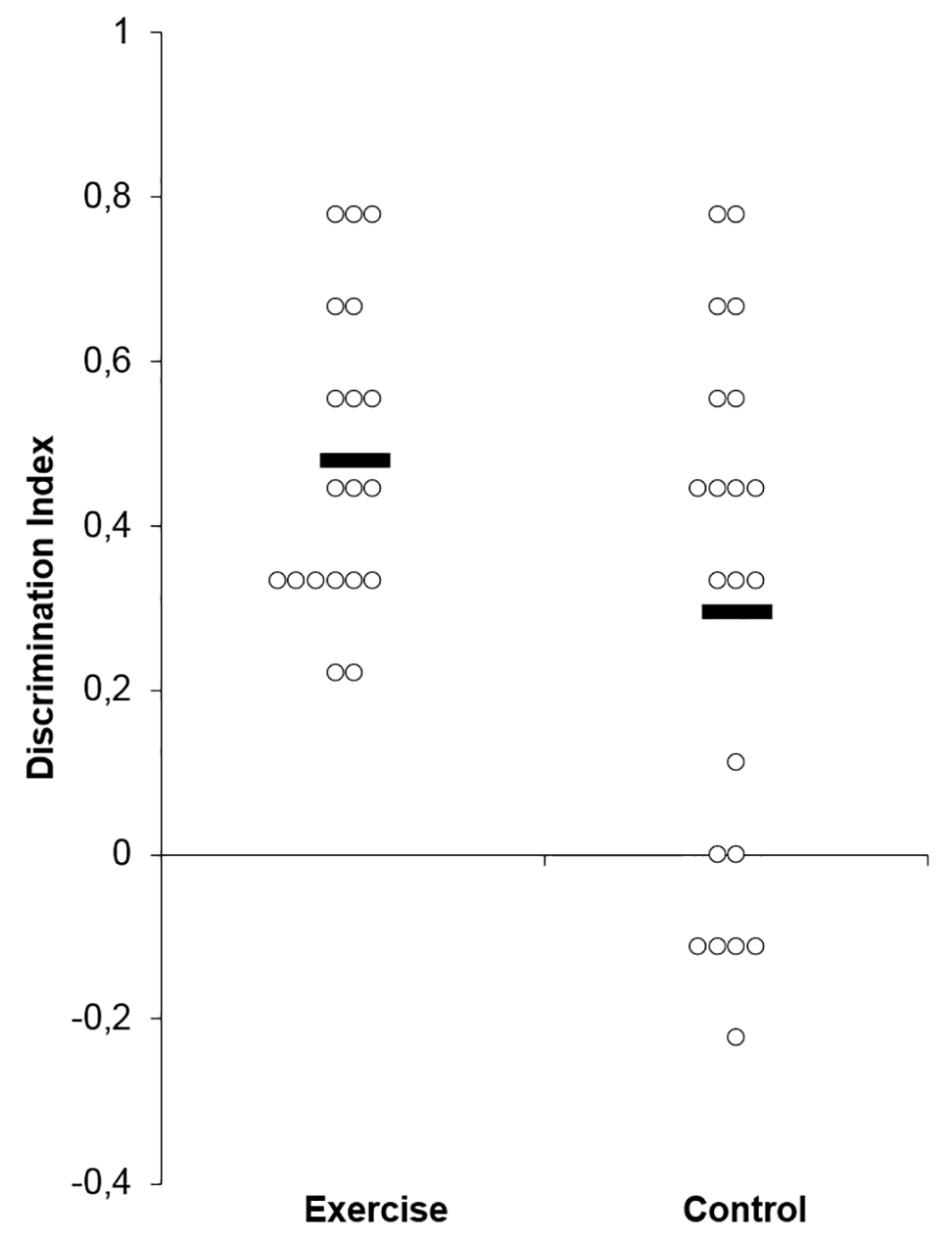
Mean and individual discrimination index scores across the exercise and control groups (Experiment 3).

Figure 3 displays the individual participant discrimination index scores across the two groups. As indicated, for experiment 3, the exercise and control groups, respectively, had a discrimination implicit memory index score of 0.48 (0.18) and 0.29 (0.32) (*t*(38) = 2.16, *p* = .03).

### Discussion

The purpose of this experiment was to extend previous work that has mostly focused on: 1) recognition memory tasks using a computerized approach for single dimension objects, and 2) the effects of exercise on explicit memory function. Specifically, we evaluated the potential effects of a moderate-intensity bout of aerobic exercise on the implicit memory recognition of 3-dimensional objects that were not explicitly encoded. Our experimental results demonstrate that a brief, moderate-intensity bout of aerobic exercise was effective in enhancing implicit memory. Notably, however, this overall enhancement effect appears to be driven by differences observed for the context-typical objects. Further, the overall differences in the discrimination index also appear to be driven by a lower false rate (vs. higher hit rate) among the exercise group (vs. control group).

Our findings align with other emerging work in this field showing that acute exercise may subserve explicit and implicit memory function (Loprinzi & Edwards, 2017; Loprinzi et al., 2018a). There are overlapping mechanisms and pathways influencing explicit and implicit memory, which have been described elsewhere (Barco et al., 2006). Acute exercise may influence both of these memory types by, for example, altering neuronal excitability and activating pathways involved in long-term potentiation and synaptic plasticity (Loprinzi & Edwards, 2017; Loprinzi et al., 2017; Loprinzi & Frith, 2019; Piepmeier & Etnier, 2015).

These findings also have numerous implications, as much of the information individuals acquire in their daily lives are done incidentally. For example, individuals are not always actively trying to memorize or encode spontaneous events that occur throughout the day, faces they encounter, or objects exposed to during the day. Fortunately, emerging work suggests that conscious perception is not mandatory for memory formation, particularly spatial-based episodic memories (Wuethrich et al., 2018). Encouragingly, our findings suggest that acute walking, which, in theory, could occur at nearly any point throughout the day, may help to facilitate the retrieval of implicit memories. An interesting follow-up experiment would be to evaluate whether acute walking, to occur during the encoding of the implicit memory (as opposed to priori to encoding, which was the case in the present experiment), may also help to facilitate implicit memory retrieval. If true, this would have greater real-world applications to the potential for exercise to enhance implicit memory, as, in theory, more implicit events are likely to occur during ambulation.

## General Discussion

Experiment 1 (lower-intensity exercise) and Experiment 2 (higher-intensity exercise) did not demonstrate any effect of acute exercise on implicit memory. However, Experiment 3 provided suggestive evidence that acute moderate-intensity exercise may improve implicit memory when assessed in a more real-world way.

Subconscious encoding of material, in theory, may be more enhanced for real-world type objects. If true, this may, in part, explain the notable differences across our experiments. Further, research demonstrates that unconscious relational encoding of multiple objects may depend on the hippocampus (Duss et al., 2014; Hannula & Greene, 2012; Reber, Luechinger, Boesiger, & Henke, 2012), a critical brain structure that is influenced (increased neuronal activity) by acute exercise (Rendeiro & Rhodes, 2018). Perceptual implicit processing may occur from priming existing memories (Kuldas, Ismail, Hashim, & Bakar, 2013). Perhaps our real-world implicit memory task was more robust in priming such memories, and thus, facilitated the role of acute exercise in such implicit processes. Clearly, additional work in this area is needed. If confirmed by future experimentation, then critical thought will be needed to evaluate the mechanism through which acute exercise may enhance implicit memory.

Limitations of these experiments is the relatively small, homogenous samples evaluated. Thus, larger samples in broader populations may help increase the generalizability of our findings. Further, although Experiment 2 employed a higher exercise intensity than Experiment 1 (20 bpm higher heart rate), this may not have been large enough of a physiological change to elicit improvements in memory function. Strengths, however, include the experimental approach of three integrated studies.

In conclusion, these novel experiments demonstrate that acute exercise does not influence a commonly used laboratory-based assessment of implicit memory but may enhance real world-related implicit memory function. Future confirmatory work on this paradigm is warranted. Such work should also evaluate the potential effects of other exercise parameters (e.g., duration, temporality, setting) on implicit memory.
